# Clinical Variability of Pediatric MERS: Insights from a Retrospective Observational Study

**DOI:** 10.3390/jcm14124169

**Published:** 2025-06-12

**Authors:** Mariaelena Romeo, Maria Polselli, Vittorio Mantero, Romina Moavero, Luigi Mazzone, Massimiliano Valeriani

**Affiliations:** 1 Child Neurology and Psychiatry Unit, Systems Medicine Department, Tor Vergata University of Rome, 00133 Rome, Italy; romina.moavero@opbg.net (R.M.); luigi.mazzone@uniroma2.it (L.M.); massimiliano.valeriani@opbg.net (M.V.); 2 Department of Neurology, ASST Lecco, 23900 Lecco, Italy; v.mantero@asst-lecco.it; 3 Developmental Neurology, Ospedale Pediatrico Bambino Gesù, IRCCS, 00165 Rome, Italy; 4 Translational Pain Neuroscience and Precision Medicine, CNAP, Department of Health Science and Technology, School of Medicine, Aalborg University, 9260 Aalborg, Denmark

**Keywords:** clinically mild encephalitis/encephalopathy with a reversible splenial lesion (MERS), splenium, reversible splenial lesion syndrome (RESLES), MRI, pediatric, corpus callosum

## Abstract

**Background/Objectives**: Mild encephalitis/encephalopathy with reversible splenial lesion (MERS) is a rare neurological disorder primarily affecting pediatric patients but also observed in adults. The radiological hallmark of MERS is a reversible lesion in the splenium of the corpus callosum. Although MERS generally has a favorable prognosis, its variable presentation poses diagnostic challenges. This study examines the clinical variability, diagnostic hurdles, and outcomes of pediatric MERS cases. **Methods**: Our retrospective study included 19 pediatric patients (11 female and 8 males with an average age of 8.41 years) diagnosed with MERS between 2016 and 2024. Clinical data, including demographic characteristics, prodromal symptoms, neurological features, MRI findings, laboratory results, treatments, and outcomes, were analyzed. **Results**: Among the 19 patients, 84% were previously healthy, with the remaining 16% having pre-existing medical conditions. The most common prodromal symptoms were fever (68%), vomiting (47%), and diarrhea (32%). Neurological manifestations included seizures (26%), headache (21%), and drowsiness (21%), among others. In terms of etiology, infections were identified in 52% of the patients, with viral agents, particularly rotavirus, being the most common (40%). Hyponatremia was present in 63% of the cohort. The typical MRI splenial lesion underwent complete resolution in all patients. Treatment varied, with 53% of patients receiving electrolyte rehydration, and 21% receiving intravenous immunoglobulin or corticosteroids. All patients, but one, achieved full recovery. **Discussion**: This study reinforces the clinical heterogeneity of MERS in pediatric patients, emphasizing its favorable prognosis independently of presentation. Viral infections and hyponatremia were the most frequent etiologies.

## 1. Introduction

Mild encephalitis/encephalopathy with reversible splenial lesion (MERS) is a rare but increasingly recognized clinico-radiological entity, particularly within pediatric populations [1]. Initially described in the early 2000s, MERS predominantly affects children, although it has been described also in adults [1].

In 2011, the term “reversible splenial lesion syndrome” (RESLES) was proposed [2] as a general term for diverse etiologies, while the term MERS was primarily referred to infection-related entities. Currently, the two terms are occasionally employed interchangeably. Recently, a new, more extensive term—cytotoxic lesions of the corpus callosum (CLOCCs)—has been used as a general description for all conditions, including MERS, RESLES, reversible splenial lesions, and transient splenial lesions [3].

MERS clinical manifestations are different and often nonspecific [4,5].

Mild encephalitis/encephalopathy with a reversible splenial lesion (MERS) is a rare neurological syndrome characterized clinically by transient encephalopathic symptoms and radiologically by a reversible lesion in the splenium of the corpus callosum, typically visible on magnetic resonance imaging. Epidemiological data are limited, mainly due to the rarity of the condition and potential underdiagnosis. However, a letter reported a radiological prevalence of approximately 3% in certain pediatric cohorts [6] suggesting that MERS may be more common than previously recognized. The syndrome has been described globally, with a higher concentration of cases reported in East Asia—particularly Japan and China—but also in Europe, Australia, and Turkey. It predominantly affects children and young adults, with a median age of around 16 years and a slight male predominance.

This condition is typically characterized by a prodromal illness consisting of fever, cough, vomiting or diarrhoea, followed by encephalopathy. Common neurological symptoms include altered mental status, seizures, headache, behavioral changes and ataxia [7]. While the condition is typically self-limiting with full recovery, its heterogeneous presentation and the evolving diagnostic criteria represent significant challenges for early diagnosis and management [8].

The radiological hallmark of MERS is a reversible lesion in the corpus callosum, identifiable by Magnetic Resonance Imaging (MRI). Specifically, MRI reveals an ovoid lesion in the mid-splenium of the corpus callosum (SCC), with high signal intensity on diffusion-weighted imaging (DWI), reduced signal intensity on apparent diffusion coefficient (ADC) maps, high signal intensity on FLAIR (fluid-attenuated inversion recovery), and no enhancement after gadolinium infusion [1]. MERS type 1 lesions are confined to the splenium, whereas MERS type 2 lesions extend into the anterior parts of the corpus callosum and/or the periventricular white matter [4,9].

The exact pathophysiology of MERS remains unclear, though several mechanisms have been proposed, including viral infections, autoimmune responses, and metabolic disturbances [7]. Some studies suggest that intramyelinic edema, caused by separation of myelin layers, interstitial edema in tightly packed fibers, and a transient inflammatory infiltrate, may play a role in an immune-mediated mechanism [8]. Other hypotheses propose that hypotonic hyponatremia may lead to water influx into the brain, causing intramyelinic edema and resulting in transient diffusion restriction observed in MRI [1,8].

Although specific treatment guidelines for MERS have not been fully established, management generally consists in supportive care, addressing issues such as seizures, dehydration, and hyponatremia [10]. Antiviral drugs, corticosteroids, and intravenous immunoglobulin (IVIg) are typically reserved for severe cases or when specific underlying causes are identified [11].

The prognosis is generally favorable, with MRI lesions resolving and clinical symptoms regressing within days to weeks [1,12]. While several case reports have improved our understanding of MERS, its clinical heterogeneity in children remains inadequately defined [1]. This variability complicates diagnosis, as MERS may resemble other encephalopathies or be misdiagnosed in favor of more common causes [7]. Moreover, despite a generally favorable outcome, the risk of misdiagnosis or delayed treatment remains a concern, particularly when symptoms overlap with more serious neurological disorders.

Our study has different aims. (1) We explore the clinical variability of MERS in a personal case series of children, examining the spectrum of presenting symptoms, diagnostic challenges, and outcomes. (2) Since most published case series include only Asian patients, we want to investigate whether the clinical, radiological, and laboratory characteristics of western children and adolescents with MERS differ or not from what is already known. In order to reach this purpose, we collected the largest sample of western young patients. (3) We seek to emphasize the importance of considering MERS in the differential diagnosis of acute encephalopathy in children, underscoring the need for heightened clinical awareness and further research to refine our understanding of the condition pathophysiology and optimal management strategies.

## 2. Materials and Methods

Our retrospective study included 19 patients seen at Ospedale Pediatrico Bambino Gesù (16 patients) and Lecco Hospital (3 patients), on both an outpatient and inpatient basis (Table 1). Eleven patients were female (57.9%) and 8 were male (42.1%). The average age was 8.41 years, with a standard deviation of 4.96 years. All patients were diagnosed with MERS between 2016 and 2024. Participants were eligible for inclusion if they were under 18 years of age and had a confirmed diagnosis of MERS (Mild Encephalitis/Encephalopathy with a Reversible Splenial lesion).

Exclusion criteria included the presence of neurodevelopmental disorders, major neurological conditions such as stroke or demyelinating diseases, brain tumors and known genetic syndromes.

Five outpatient cases had previously been diagnosed with MERS in other healthcare institutions and were subsequently followed up at our hospital as part of a comprehensive care management plan. Clinical data, including demographic characteristics, prodromal and neurologic symptoms, neurologic examination, MRI and electroencephalography findings, laboratory findings, treatment, and prognosis were reviewed retrospectively.

## 3. Results

### 3.1. Preexisting Illness

Seventeen patients (89%) were previously healthy. Two patients (11%) had already been diagnosed with preexisting illnesses: sleep disorder and migraine (1 patient—5%), aortic coarctation and heart failure (1 patient—5%).

### 3.2. Prodromal Symptoms

Thirteen patients had prodromal symptoms such as fever (68%), vomiting (47%), diarrhea (32%), reduced appetite (21%), asthenia (16%), weight loss (11%), abdominal pain (5%), dehydration (5%), petechiae (5%), conjunctivitis (5%), and skin rash (5%) No prodromal symptom was detected in 2 patient (11%).

### 3.3. Neurological Manifestations

**Figure 1:**
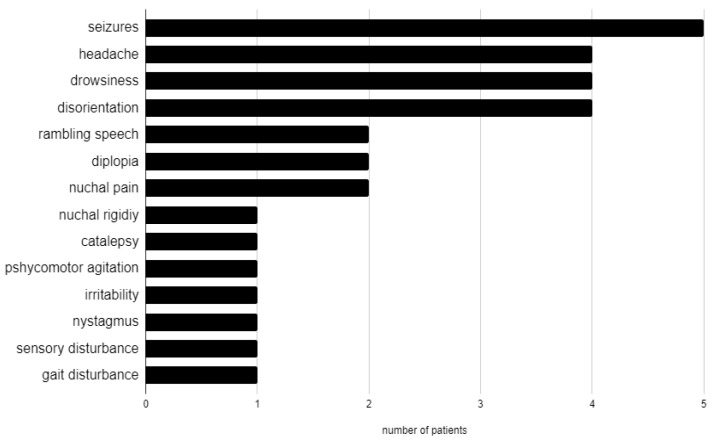
Neurological manifestations in our cohort.

Neurological manifestations (Figure 1) were: seizures in 5 patients (26%), headache in 4 (21%), drowsiness in 4 (21%), disorientation in 4 (21%), rambling speech in 2 (11%), diplopia in 2 (11%), nuchal pain in 2 (11%), nuchal rigidity in 1 (5%), catalepsy in 1 (5%), psychomotor agitation in 1 (5%), irritability in 1 (5%), bilateral nystagmus in 1 (5%), sensory disturbance in 1 (5%), and gait disturbance in 1 (5%). Three patients had no neurological manifestation (16%).

### 3.4. Putative Etiology

As shown in Table 2, infectious agents were detected in 10 patients (52%), while noninfectious causes were identified in 3 (16%), In 6 patients (32%), no putative etiology could be demonstrated. Among infectious agents, viral infections were detected in 7 out of 10 patients (70%). Particularly, Rotavirus was isolated from stool samples in 4 patients (40%), HHV-6 was identified in 2 patients (20%) through blood specimen, and Parvovirus B19 was detected in 1 patient (10%) from cerebrospinal fluid (CSF) samples. Bacterial infections were detected in 3 patients (30%). Particularly, *E. coli* was isolated from stool samples in 1 patient (10%), Haemophilus was identified in 1 patient (10%) from tracheal aspirate samples, suspected diagnosis of Meningococcus sepsis was made in 1 patient on the clinical basis (10%), even though the Meningococcus itself was not isolated. Hyponatremia was detected in 12 patients (63%): in 8 patients it was associated with an infectious agent and in 2 patients (11%) with Multisystem Inflammatory Syndrome in Children (MIS-C). In 2 patients (11%), hyponatremia represented an isolated finding.

### 3.5. Instrumental Examination

**Figure 2:**
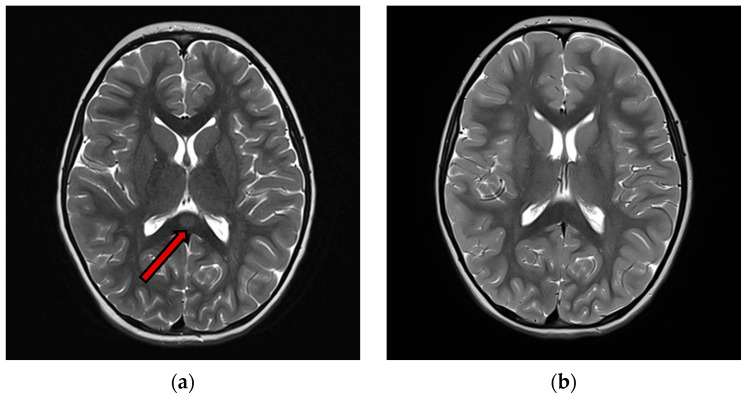
(**a**) T2 images of acute phase of MERS Type 1 (**b**) T2 weighted images of the same patient of acute phase of MERS type 1. An ovoid high signal area is observed in the midmedulla of the body of the corpus callosum (arrow), 3 months after the previous, showing complete resolution of the lesion.

Hyponatremia was found in 12 patients (63%), normal sodium levels in 7 patients (37%). C-reactive protein (CRP) was high in 13 patients (68%). White blood cells were high in 5 patients (26%). Only 9 patients underwent lumbar puncture, and CSF cell count was normal for all of them (100%). An EEG recording was performed in 14 patients. Nine patients (64%) showed an abnormal EEG. Almost half of these patients (4/9 cases) exhibited a slow background activity (BA). In two patients, EEG showed epileptic anomalies and in four EEG recordings there were slow anomalies, without clear epileptic abnormalities. A contrast-enhanced brain MRI with T1, T2-weighted sequences, DWI and FLAIR studies at the onset was performed in all patients. Eighteen patients (95%) showed type I lesions (Figure 2), while 1 patient (5%) had type II lesions. A follow-up MRI was available in 13 patients within a time interval ranging from 1 week to 10 months from the acute episode. In all these cases, the follow-up contrst-enhanced MRI showed complete resolution of the previously observed lesions, including on the T1, T2-weighted sequences DWI and FLAIR studies.

### 3.6. Treatment and Outcome

As shown in Table 3, glucoelectrolytic rehydration was performed in 10 patients (53%); in 4 cases, it represented the only treatment. Antibiotics were administered to 5 patients (26%), intravenous immunoglobulin (IVIg) to 4 (21%), corticosteroids to 4 (21%), antiviral drugs to 3 (16%), and immunosuppressant drugs (Eculizumab and Anakinra) in 2 (11%). Two children, who presented with seizures, had antiepileptic medications. In 5 patients no pharmacological treatment was administered (26%). While 13 patients recovered completely, only 1 patient had sequelae after the acute event. This patient was the only one in our cohort to experience a convulsive status epilepticus. She received antiepileptic drugs and corticosteroids, and the follow-up brain MRI performed one week after onset showed a complete resolution of splenial lesion. However, she continued to show motor and speech difficulties for other 6 months.

## 4. Discussion

This study contributes to the growing body of literature on MERS in youth, with key findings that offer valuable insights concerning its clinical presentation and potential pathophysiology. The main findings from our cohort include: (1) heterogeneity in clinical manifestations, with a wide range of neurological and prodromal, particularly gastrointestinal, symptoms; (2) heterogeneity in potential causes, as we identified both viral and bacterial infections as triggers for MERS, with rotavirus being the most frequent viral agent; and (3) a favorable outcome for most patients, with nearly all children experiencing full recovery.

Most previous studies on pediatric MERS were based on Asian cohorts [5,13,14,15,16,17,18,19,20,21], with western studies on MERS in children being limited to a few case reports and small case series [22,23,24,25,26,27,28,29,30,31]. To the best of our knowledge, our study represents the largest western population with pediatric MERS.

In a recent review by Chen et al. [21], it was reported that more than 60% of the cases of MERS aged under 3 years. The authors suggested that MERS is more likely to occur in young children due to the immaturity of their central nervous system and their increased susceptibility to infections. In our case series, only 4 patients were under the age of 3, while the majority of the population was older.

It is important to note that almost all of our patients were healthy prior to the diagnosis of MERS, with the exception of two cases. The first was an infant who was incidentally diagnosed with MERS during a multidisciplinary follow-up after surgery for aortic coarctation. This finding suggests that, while MERS typically affects previously healthy children, underlying medical conditions or recent surgical interventions could play a role in the development of the disease.

In literature, infections are commonly identified as the most frequent cause of MERS [32]. Our findings are in agreement, supporting the hypothesis that infectious agents may play a role in the immune mechanism contributing to MERS development. Various infectious agents have been linked to MERS in children, including rotavirus, adenovirus, influenza A and B, Epstein-Barr virus, Dengue virus, mumps virus, herpes simplex virus, parainfluenza, parvovirus B19, cytomegalovirus, Mycoplasma pneumoniae, Streptococcus pneumoniae, and Campylobacter jejuni [5]. In our study, viral infections were identified in 7 of 10 infectious cases, with rotavirus being the most frequent agent (4 patients). Additionally, Human Herpesvirus 6 (HHV-6) and Parvovirus B19 were also implicated. Bacterial infections were detected in 3 patients, consistent with prior research showing that bacterial pathogens, though less common, can contribute to MERS [32]. Hyponatremia has been proposed as a potential cause of MERS. In our cohort, the percentage of patients with hyponatremia was 63% (12 out of 19 patients). Diarrhea and vomiting may also contribute to sodium imbalances, as well as the effects of dehydration. The association between hyponatremia and infections in 8 patients supports the hypothesis that electrolyte disturbances may result from the infectious process or the associated inflammatory response. Two patients of ours had overlapping diagnoses of MERS and MIS-C, with a positive serology for SARS-CoV-2 (anti-N and anti-S antibodies), consistent with a previous viral infection. In pediatric patients, MIS-C is a clinical manifestation within the clinical spectrum of COVID-19 [33]. After assessing the radiological outcomes of 45 pediatric patients with MIS-C, Palabiyik et al. [34] showed that the most common finding on brain MRI was MERS. This suggests that in patients with a suspected MERS diagnosis SARS-CoV-2 antigen and antibody tests should be included to evaluate the potential overlap with MIS-C.

The most frequent prodromal symptoms in pediatric patients later diagnosed with MERS are fever, vomiting, diarrhea, abdominal pain, and headache [5]. The prodromal symptoms observed in our patients were generally in agreement with those reported in the literature, with a predominance of fever and gastrointestinal symptoms. As for the neurological symptoms, alterations of consciousness, seizures, drowsiness, headache, monoparesis, abnormal speech, visual hallucinations, and ataxia have been more often reported in the literature. In our cohort, seizures represented the most frequent neurological symptom. Alterations of consciousness showed a higher frequency than that previously reported. Drowsiness, headache, and other neurological symptoms (e.g., abnormal speech, diplopia, and psychomotor agitation) were observed in a significant proportion of patients. The heterogeneous neurological symptoms suggest that MERS can affect different brain networks, with some patients experiencing more localized symptoms and others presenting with global encephalopathy.

As for the instrumental examinations, absence of pleocytosis in the cerebrospinal fluid (CSF) in all our patients supports the hypothesis that MERS is an infection-associated encephalopathy syndrome rather than an infectious encephalitis. In our study, 64% of the patients who underwent EEG showed abnormal results. This is in agreement with the systematic review by Chen et al. [21], reporting an abnormal EEG in 60% of patients with MERS. In our patients, brain MRI showed more frequent Type I than Type II lesions, thus confirming previous findings [5,21].

Regarding treatment, there are no established guidelines for the treatment of MERS [5]. Pulsed therapy with methylprednisolone and IVIg is recommended for patients with infectious encephalopathy, regardless of the pathogen or clinical-radiological syndromes [35], as well as electrolyte correction in case of electrolyte imbalances. A previous review [20] has reported that most patients with MERS could have a full recovery regardless of treatment, suggesting that specific treatments may not be necessary. In our population, the most common treatment was electrolyte rehydration, followed by symptomatic treatment with antibiotics, IVIg, and corticosteroids. Five patients received glucose-electrolyte rehydration without additional medications, while two cases did not receive any treatment. Since pharmacological treatment may be useless, depending on the clinical manifestations, an early diagnosis of MERS could avoid invasive therapies.

In the literature, most pediatric MERS cases report complete recovery without sequelae [15,21]. A case with sequelae was reported by Chen et al. [15], who described a patient with intellectual disability despite normal follow-up brain MRI. While a pediatric case of Type I MERS, following H1N1 influenza virus infection, showed persisting intellectual disability after splenial lesion resolution [36], Type II lesions are more likely associated to neurological sequelae [5]. In our pediatric population, all patients had complete clinical resolution, except for one patient who experienced movement and speech difficulties recovered after a 6-months rehabilitative treatment. In this patient, brain MRI at onset had showed a Type I lesion which had completely recovered after one week.

### Limitations of the Present Study and Future Perspectives

Our study has several limitations. First, it is retrospective, which may have introduced which may have introduced both recall and selection bias. Clinical data such as EEG findings, CSF analysis, and follow-up assessments were not uniformly available for all patients, reflecting variability in clinical management across centers and over time. This inconsistency may have influenced our ability to identify certain patterns or associations, particularly regarding long-term neurological outcomes. Secondly, the sample size was relatively small. Lastly, the lack of a standardized follow-up protocol limited our ability to assess the long-term neurological outcomes more comprehensively. Additionally, the absence of a comparative control group, such as pediatric encephalitis patients without splenial lesions, limits our ability to definitively identify features specific to MERS. We have explicitly acknowledged this as a limitation, which should be addressed in future prospective studies with appropriate control cohorts.

We can speculate that some updated techniques of CSF examination may provide important information about MERS pathophysiology. In a retrospective study, enrolling four children with MERS and 14 age-matched healthy subjects, significantly higher levels of pNf-H (phosphorylated neurofilament heavy chain) in the CSF were observed in the MERS cases compared to controls [37]. Neurofilaments are a major structural component of neurons and consist of three subunits: a light chain, a medium chain, and a heavy chain. PNf-H levels in CSF can be used as specific biomarkers for axonal injury or degeneration. Motobayashi et al.’s [37] findings suggest that patients with MERS experience axonal damage, which is often transitory, but can explain the rare cases of MERS with evident clinical sequelae. In our opinion, the study of CSF neurofilaments in larger multicentric studies could shade light about the pathophysiology of the disease. Another important field of research could be represented by the metabolic modifications associated with MERS. From this point of view, spectroscopic MRI (sMRI) of the brain might be a useful examination in patients with MERS. Recent studies have utilized advanced MRI techniques, including MR spectroscopy (MRS), to investigate the characteristics of reversible splenial lesions. Ueda et al. [38] reported on a case of mild encephalopathy where MRS revealed slightly elevated choline levels, suggesting increased glial cell membrane turnover without neuronal damage. The signal abnormalities persisted for 90 days, indicating that such lesions may not always be rapidly reversible. Similarly, Lin and Yu [39] described a patient with a reversible focal splenial lesion associated with staphylococcal meningitis, where MRS showed relatively elevated lactate levels, highlighting metabolic disturbances as a potential underlying mechanism. Additionally, Shimizu et al. [40] conducted extensive neuroimaging of a transient splenial lesion, emphasizing the importance of comprehensive imaging in understanding the pathophysiology of such lesions. These studies underscore the utility of MRS and advanced MRI in elucidating the metabolic and structural aspects of reversible splenial lesions, providing valuable insights into their diagnosis and management.

## Figures and Tables

**Table 1:** Demographic characteristics, clinical characteristics and instrumental findings of our patients.

N°	Age Onset (Y)/ Sex	Pre Existing Illness	Etiology	Prodromal Manifestations	Neurological Manifestation	Na (136–145 mEq/L)	CRP (<0.50 mg/dL)	WBC 10^3^/L (4.00–13.50)	CSF Cell Count	Time to First MRI After the Onset (Days)	Lesion Type (1, 2)	EEG Findings	Treatment	Time to Follow-Up MRI (Days-Months)	Hospital Stay (Days)	Prognosis
1.	1 y/F	None	Rotavirus, hyponatremia	Vomiting, diarrhea, asthenia	Gait disturbance, seizure	134	0.71	14.61	Normal	1	1	Normal	Glucoelectrolytic rehydration	NE	3	CR
2.	4 m/F	Aortic coarctation, heart failure	Unknown	None, occasional finding	None	133	0.12	8.67	NE	70	1	NE	NE	1 m	15	CR
3.	15 y/F	None	HHV-6; hyponatremia	Fever, vomiting	Headache and psychomotor agitation and drowsiness	134	0.05	14.35	Normal	5	1	Slow-wave abn with a bilateral fronto-central expression	Glucoelectrolytic rehydration, antibiotics, antivirals, oral NaCl and sodium bicarbonate	22 d	63	CR
4.	2 y/F	None	Unknown	Lack of appetite and abdominal pain	Drowsiness, non-convulsive status epilepticus	137	Unknown	Unknown	Normal	3	1	Slow BA, slow sharp waves on the posterior leads with a prevalence on the right hemisphere	Antibiotics, corticosteroids, ASM	7 d	25	Movement and speech difficulties
5.	6 y/M	None	Unknown	Fever	None	138	Unknown	Unknown	NE	Unknown	1	CT abn predominant on the right hemisphere	NE	NE	16	CR
6.	16 y/M	None	HHV-6	Asthenia and low-grade fever	Headache, drowsiness and diplopia	138	0.50	5.67	Normal	2	2	Normal	Antibiotics, IVIg	3 m	16	CR
7.	4 y/F	None	Unknown	Vomiting, dehydration	Seizure	136	0.50	8.75	Normal	1	1	Normal	Glucoelectrolytic rehydration, BDZ, ASM	3 m	4	CR
8.	1 m/F	None	Haemophilus influenzae, hyponatremia	Vomiting, lack of appetite, weight loss	Drowsiness, irritability	114	6.25	9.11	NE	4	1	NE	Glucoelectrolytic rehydration, antibiotic	1 m	25	CR
9.	14 y/M	None	Sepsis of suspected meningococcal origin, bacterial meningitis, hypertransaminasemia	Fever, petechiae	Headache, nuchal pain and rigidity	136	28.9	20.5	Normal	1	1	NE	Glucoelectrolytic rehydration, antibiotics, antivirals, corticosteroids	3 d	10	CR
10.	5 y/F	None	Hyponatremia	Fever	Seizures	134	18.52	12.17	NE	3	1	Slow BA	Glucoelectrolytic rehydration, antivirals	NE	7	CR
11.	7 y/F	Coexisting Hemolytic-Uremic Syndrome	*E. coli* O26, hyponatremia	Diarrhea, fever	Disorientation, diplopia and bilateral nystagmus	135	2.07	6.45	NE	0	1	Slow BA and slow abn	Immunosuppressant	2 m	17	CR
12.	14 y/F	Coexisting MIS-C and acute adrenal crisis	MIS-C, hyponatremia	Diarrhea, fever, vomiting, asthenia	Catalepsy	126	37.49	14.690	Normal	2	1	Diffuse slow abn predominant in the anterior regions	Glucoelectrolytic rehydration, corticosteroids, IVIg	1 m	16	CR
13.	17 y/M	Sleep disorder, migraine	Unknown	None (occasional finding)	None	136	Unknown	Unknown	NE	Unknown	1	NE	NE	Unknown	Unknown	CR
14.	10 y/F	None	Unknown	Fever	Headache and nuchal pain	136	Unknwn	Unknown	Normal	Unknown	1	Unknown	NE	1 m	Unknown	CR
15.	7 y/M	None	Parvovirus B19, hyponatremia	Fever, vomiting	Seizures	132	8.03	17.98	Normal	1	1	Sporadic slow waves in the left centro-parietal region	IVIg	4 m	4	CR
16.	9 y/M	None	MIS-C, hyponatremia	Fever, vomiting, conjunctivitis and skin rash	Sensory disturbance	126	16.62	5.06	NE	1	1	Slow BA	IVIg, corticosteroids, immunosuppressant	NE	19	CR
17.	8 y/M	None	Rotavirus, hyponatremia	Diarrhea, fever	Disorientation and poorly reactive	130	0.88	12.6	NE	1	1	Normal	Glucoelectrolytic rehydration	3 m	Unknown	CR
18.	8 y/F	None	Rotavirus, hyponatremia	Diarrhea, fever, vomiting, lack of appetite, weight loss	Disorientation, rambling speech	122	9.08	11.0	NE	1	1	Slow abn on the frontotemporal regions bilaterally	Glucoelectrolytic rehydration	NE	Unknown	CR
19.	8 y/M	None	Rotavirus, hyponatremia	Diarrhea, fever, vomiting, lack of appetite	Disorientation, rambling speech	127	7.52	8.4	Normal	1	1	Normal	Glucoelectrolytic rehydration	4 m	Unknown	CR

M = male; F = female; y = years; NE = not examined; MIS-C = Multisystem Inflammatory Syndrome in Children; BA = background activity; ASM = anti seizure medication; IVIg = intravenous immunoglobulin; CR = complete recovery; CT = centrotemporal; abn = abnormalities.

**Table 2:** Putative etiologies in our patients.

**Pathogen Infection**	**Data *n***	**%**
Rotavirus	4/10	40
HHV-6	2/10	20
Parvovirus B19	1/10	10
*E. coli*	1/10	10
*H. Influenzae*	1/10	10
Meningococcus	1/10	10
**Noninfectious causes**	**Data *n***	**%**
Hyponatriemia	12/19	63
MIS-C	2/19	11

**Table 3:** Treatment in our patients.

Treatment	Data *n*	%	Posology
Glucoelectrolytic rehydration	10/19	53	Hollyday-Segar Formula
Antibiotics	5/19	26	Weight-based
IVIg	4/19	21	A single dose of 2 g/kg
Corticosteroids	4/19	21	Weight-based
Antiviral drugs	3/19	16	Weight-based
Immunosuppresant drugs	2/19	11	Weight-based
Antiepileptic medications	2/19	11	Weight-based
No treatment	5/19	26	-

## Data Availability

The data are extracted from our patients’ medical records, therefore they cannot be shared due to privacy reasons.

## Abbreviations

The following abbreviations are used in this manuscript:

MERS	Mild encephalitis/encephalopathy with reversible splenial lesion
MRI	Magnetic Resonance Imaging
RESLES	Reversible splenial lesion syndrome
CLOCCs	cytotoxic lesions of the corpus callosum
SCC	splenium of the corpus callosum
DWI	diffusion-weighted imaging
ADC	apparent diffusion coefficient
FLAIR	fluid-attenuated inversion recovery
IVIg	intravenous immunoglobulin
MIS-C	Multisystem Inflammatory Syndrome in Children
HHV-6	Human Herpesvirus 6
CSF	cerebrospinal fluid
pNf-H	phosphorylated neurofilament heavy chain
sMRI	spectroscopic MRI
